# Factors that influence general practitioners' obesity‐related clinical practices and determinants of behavior to target to promote best practice in obesity care: A qualitative exploration

**DOI:** 10.1002/osp4.70012

**Published:** 2024-09-28

**Authors:** Leona Ryan, Grainne O’Donoghue, Michael Crotty, Susie Birney, Caroline Heary, Michelle Hanlon, Owen Conlan, Jane C. Walsh

**Affiliations:** ^1^ School of Psychology University of Galway Galway Ireland; ^2^ School of Public Health, Physiotherapy and Sports Science University College Dublin Dublin Ireland; ^3^ Irish College of General Practitioners (ICGP) Clinical Lead Obesity Dublin Ireland; ^4^ Irish Coalition for People Living with Obesity (ICPO) Dublin Ireland; ^5^ School of Computer Science and Statistics Trinity College Dublin Dublin Ireland

**Keywords:** behavior change, general practice, obesity, qualitative

## Abstract

**Introduction:**

General practitioners (GPs) have been identified as pivotal in the identification and initiation of treatment for obesity, yet effective obesity management remains challenging in general practice. Despite the growing prevalence of obesity and the central role of GPs, there is a dearth of research exploring their experiences and challenges in managing the disease.

**Objective:**

This study aimed to understand these challenges by exploring GPs' experiences and to identify factors influencing their obesity management practices to inform the development of targeted intervention strategies.

**Method:**

In‐depth interviews were conducted with 10 GPs. Data were analyzed using abductive thematic analysis underpinned by the theoretical domains framework (TDF). Findings were mapped to the behavior change wheel (BCW) and the behavior change taxonomy to identify potential future intervention strategies.

**Findings:**

Participants described multiple barriers to effective obesity management, including knowledge gaps, lack of training, patient factors, and systemic challenges. Key themes identified were the need for increased support, improved patient engagement, and system‐level changes.

**Conclusion:**

This study offers valuable insights into the challenges GPs encounter when managing obesity in general practice. Application of the TDF and BCW frameworks identified complex interactions between knowledge, beliefs, and environmental factors influencing GP behavior. These findings highlight key areas for targeted interventions to enhance obesity care and drive best practice.

## Graphical abstract

A qualitative study explored general practitioners' (GPs') influences on obesity management practices and identified potential targets for intervention. The Theoretical Domains Framework and Behavior Change Wheel informed the analysis, highlighting the influence of multiple factors on current practices. The findings suggest that future interventions should target GPs' knowledge and skills and the broader clinical environment to promote best practices in obesity management. Collaborative development of interventions with GPs is recommended to ensure optimal impact.

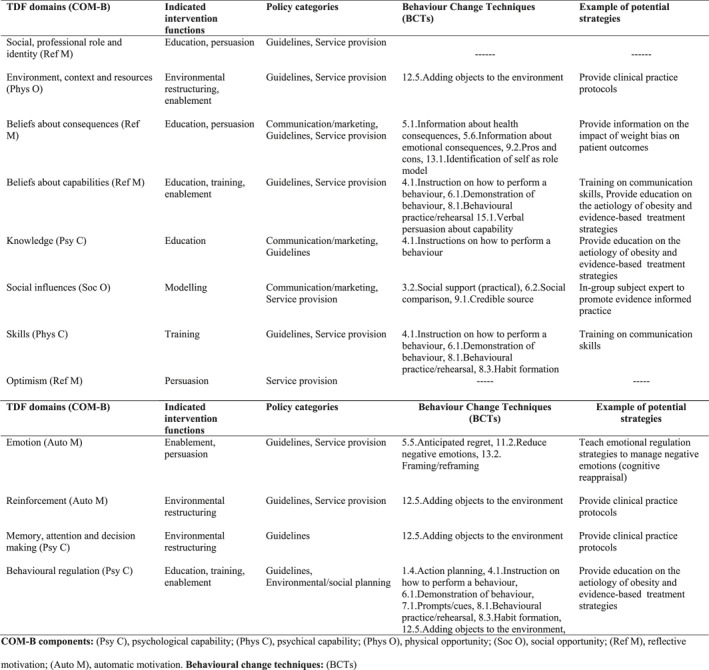

## 1 INTRODUCTION

Global projections estimate that by 2030, 1 in 5 women and 1 in 7 men will be living with obesity.^1^ Ireland is projected to experience a significant increase in obesity rates within Europe, with predictions of 48% for males and 57% for females by 2030.^2^ Consequently, a significant surge in obesity‐related illnesses, including coronary heart disease, stroke, cancers, and type 2 diabetes, is anticipated. This is expected to result in an estimated medical expenditure of €5.4 billion by 2030.^2^ In response to the rising prevalence of obesity, the Health Service Executive in Ireland introduced the Model of Care for the management of overweight and obesity (MOC).^3^ The MOC outlined a tiered approach to obesity management, emphasizing early intervention in general practice and escalation to specialist services as the complexity of the disease increased.^3^ General practitioners (GPs) were identified as pivotal in identifying and initiating treatment options for obesity.^4^

However, previous research indicated a significant gap between the expected role of GPs in obesity management and their perceived preparedness.^5^ A subsequent analysis of electronic patient records (*N* = 91, 413) confirmed this discrepancy, revealing a substantial underutilization of weight management interventions among patients with obesity (PwO) in general practice.^6^ The study found that 90% of patients received no recorded weight management interventions. While lifestyle advice was commonly offered to those with lower levels of obesity, patients with severe obesity often lacked access to appropriate care. Furthermore, weight loss progress was rarely monitored.^6^ These findings aligned with previous research attributing this disparity to factors such as time constraints, resource limitations, and a limited understanding of obesity etiology and environmental determinants.^5^, ^6^, ^7^

The overarching objective of the MOC was to ensure equitable and timely access to appropriate healthcare services for individuals impacted by overweight and obesity.^3^ A core component involved fostering a non‐stigmatizing environment within the healthcare system. To accomplish these objectives, the MOC emphasized the need for enhanced education and training for healthcare providers (HCPs). Specifically, the MOC recommended optimizing existing obesity‐related clinical practices through targeted educational and training initiatives for HCPs.^3^

Previous research indicated a limited understanding of obesity management within general practice.^6^, ^8^, ^9^ GPs often perceived obesity as a refractory lifestyle condition, frequently reflected in stigmatizing behaviors^10^, ^11^ toward PwO, including patronizing attitudes, poor communication, and an excessive focus on weight loss.^12^, ^13^ These unaddressed attitudes toward obesity may hinder the successful adoption and implementation of future education and training interventions.^1^, ^14^ Therefore, to effectively implement new obesity‐related clinical practices, it is essential to identify the underlying attitudes, instrumental goals and contextual factors influencing current obesity management practices from the perspective of GPs.^15^, ^16^

The use of behavioral science in understanding factors that influence the behavior of healthcare providers has been shown to improve the effectiveness of interventions.^17^ Theoretical Domains Framework (TDF) and behavior change wheel (BCW) provides a robust foundation for this research.^17^ The TDF categorizes influences on behavior into 14 domains, including knowledge, beliefs, and social factors. The BCW outlines three conditions for behavior change (capability, opportunity, and motivation; COM‐B) and links them to potential intervention functions and policy categories to support the delivery of interventions.^18^, ^19^, ^20^ The model accounts for environmental influences, individual differences, intrapsychic variables, and group dynamics that motivate behavior change.^19^, ^20^ The COM‐B components link to TDF domains, which detail specific influences to target in an intervention, making the model beneficial in the creation of interventions due to its empirically based predictive power.^18^

Previous research has successfully applied the TDF and BCW to identify factors influencing healthcare provider behavior across various clinical settings.^21^, ^22^, ^23^ However, these frameworks have not been used to explore GPs' perspectives on obesity management. To address this gap, this study utilized the TDF and BCW to comprehensively understand the factors shaping GPs' obesity‐related practices and to inform the development of targeted interventions.

## 2 METHOD

The study was reported in accordance with the Consolidated Criteria for Reporting Qualitative Research (COREQ) guidelines^24^ (Table S1). Ethical approval was granted for this study by the University of Galway Research Ethics Committee (approval reference number: 2021.11.021).

### 2.1 Study design

A qualitative abductive approach based on abductive thematic analysis outlined by Thompson^25^ was used to collate and theorize the collected data. Abductive research originates from the philosophical field of pragmatism, the central tenet of which is that knowledge and reality are derived from socially constructed beliefs and habits, and that those beliefs should be evaluated on their practical implications.^26^ In practice, abductive thematic analysis is not exclusively aligned with inductive or deductive methodologies, instead it encouraged equal and parallel engagement with empirical data and existing theoretical understanding to find the most useful explanation for the phenomenon being explored.^25^, ^27^ This approach enabled the re‐examination of the empirical data in relation to the existing theoretical literature while keeping the participants insights at the core of theme generation.^28^

### 2.2 Participants

Ten GPs practicing in Ireland participated in this study. Participants were recruited using purposive and snowball sampling to produce representation across sex, age, and experience levels. Invitations were emailed to GPs through the Primary Care Trials Network. The following contact from interested participants, study information packs were provided (study overview, data protection and consent forms). No participants dropped out of the study. The study used information power to determine sample size, considering factors such as the study's focused aims, specificity of the sample, theoretical underpinnings, rigorous data analysis and the quality of the data collected.^29^ Information power was chosen over data saturation as it prioritized the collection of rich meaningful data over the quantity of respondents. While saturation ensures a baseline level of data completeness, it does not guarantee a rich or nuanced understanding. Conversely, information power prioritized the quality and depth of the information collected. Reaching information power means that the collected data provided sufficient insightful and meaningful information to answer the research questions.^29^

### 2.3 Data collection

A topic guide was developed considering the validated version of the TDF^18^ (Table S2) and the broader literature related to the study. It was refined after piloting with an experienced health psychologist with expertise in using the TDF in qualitative research. The guide was used adaptively to facilitate a conversational flow exploring the potential factors that influence GPs' obesity‐related clinical practices.^30^ Individual semi‐structured interviews were conducted online via Zoom video‐conferencing between September—December 2022; interviews lasted between 25 and 60 min (*M* = 34). Field notes were recorded after each interview. To ensure consistency one author (LR) conducted the interviews. The interviews were digitally recorded with the participants' consent and transcribed verbatim. The transcriptions were imported in to NVivo 20 for analysis.^31^

### 2.4 Data analysis

The analytical process was based on abductive thematic analysis.^25^ The lead author familiarized herself with the transcriptions. Notes were taken that contextualized meaningful patterns identified across the collected data. Inductive line‐by‐line coding at the semantic level was conducted, iteratively refined and organized into initial conceptual clusters. A codebook was generated to provide structure to the coding process. In keeping with an abductive approach, the codebook was used to enhance reflection on the process, and as a tool to prompt shared‐meaning discussion with the research team. Data was then analyzed through the lens of the TDF and deductive changes were made to the coding structure either through additions or updates. The coding structure was amalgamated, recontextualised and organized into themes. In the next step, analysis focused on reviewing the themes in the context of the TDF, providing insight into potential underlying mechanisms that explained the empirical findings. Finally, the themes were organized and labeled as relevant constructs within the TDF, the findings of which are presented below. An overview of the abductive thematic analysis can be found in Figure S1.

## 3 RESULTS

Ten GPs participated in the study. Participants were predominantly female (60%) and based in urban areas (60%). GP experience varied, with 40% having less than 5 years, 40% having 10 years, and 20% having over 20 years of experience. Ten TDF domains influenced GPs' obesity‐related clinical practices: knowledge, environmental context and resources, skills, emotion, beliefs about consequences, beliefs about capabilities, reinforcement, social influences, professional role and identity, and behavioral regulation.

## 4 KNOWLEDGE

### 4.1 Knowledge gaps hinder obesity management

Most participants reported a limited understanding of obesity as a complex disease. Insufficient education and training on current obesity research and best practice guidelines contributed to this knowledge gap. Consequently, obesity was often perceived and treated as primarily a lifestyle issue rather than a multifaceted condition. This oversimplified view of obesity, participants suggested, led to a pessimistic outlook on patient outcomes. Participants also highlighted significant gaps in their knowledge of treatment options. This included prescribing and monitoring pharmacological interventions as well as understanding surgical options. Recognizing these knowledge deficiencies, participants acknowledged their negative impact on obesity management in general practice. A strong preference for structured treatment approaches was evident among participants. They emphasized the need for accessible, practical resources, such as evidence‐based protocols, to guide clinical decision‐making. As one GP stated, *“Don't give me a policy document. Give me an actual protocol that I can use in my practice with my patients, that's evidence‐based, that's standardized […] give me what I can do on the ground.”* Furthermore, awareness of the existing Model of Care (MOC) was limited to 80% of participants, highlighting the need for clear and focused communication strategies to promote its implementation.

## 5 ENVIRONMENTAL CONTEXT AND RESOURCES

### 5.1 Gaps in educational and training

A key contextual factor was that obesity was not prioritized within medical education and training. Obesity was reportedly framed as a risk factor for other diseases and not as a complex condition in its own right. Furthermore, this perspective was presented unsympathetically, situating blame on lifestyle factors as the leading cause of obesity. This limited perspective reportedly persisted throughout GP training. Several participants noted having to actively seek out additional, specialized obesity training that was not included in the standard curriculum: *We were never particularly taught on it as a topic as such in medical school […] you might learn about it in general practice training but mainly just on obesity being a risk factor*. *(GP, M).*

### 5.2 Lack of resources impact on continuity of care

GPs reported a lack of readily available resources as a significant barrier to obesity management. This created structural limitations, restricting treatment options for both patients and providers. Participants emphasized difficulties in accessing resources for patient support, often resorting to commercial weight loss programs due to insufficient allied health services. GPs expressed a desire for “in‐house” interdisciplinary health services to support them in delivering timely and optimal care for PwO: *I can't be referring them to a dietitian, to talk therapy, and an endocrinologist, or whatever I have to pick or what I can address. So, if there is an infrastructure, then it should be a one‐stop shop that offers all of those in one place (GP,M)*.

## 6 SKILLS

### 6.1 Weight‐based conversation training is required

Participants reported significant challenges in initiating weight‐based conversations with patients. Due to the absence of standardized guidelines, many GPs have described a trial‐and‐error approach to navigate this sensitive topic. While some participants found success in engaging patients proactively, others reported that patients were more likely to initiate these discussions themselves. Conversely, participants identified a need for specific training on how and when to initiate weight‐based conversations. Fear of provoking a negative patient response often led to avoidance of the topic: *I think training on how to have difficult conversations is uh, always lacking, and you can never do enough of it. Um![sic] And most of us learn on the job. (GP,F)*.

## 7 EMOTION

### 7.1 Fear and frustration

Participants described having an inherent fear of offending patients. This is closely aligned to *skills* where GPs avoided initiating the conversation as they felt they were not equipped to engage effectively with the patient and thus were reluctant to cause the patient any “*stress*.” However, a clear conflict was apparent between wanting to avoid upsetting patients and recognizing the importance of addressing weight for their health. Limited access to allied health support exacerbated these challenges. Participants felt disempowered by the reliance on overly simplistic weight management strategies, and described the experience as frustrating for both themselves and patients. As one GP stated, *It's important that we have those conversations with patients, but its disempowering then when they ask you, How? What do we do about it? and you say, go online do a weight loss course, or do to weight watchers, or operation transformation, or go to the gym. (GP,F)*.

## 8 BELIEFS ABOUT CONSEQUENCES

### 8.1 The pros and cons of classifying obesity as a disease

Participants held diverse views on classifying obesity as a disease. While many recognized the potential benefits for patients, including reducing stigma and facilitating treatment seeking, others expressed concerns about over‐medicalization and potential negative connotations. Some participants theorized that disease classification could empower patients, while others believed it might be disempowering. One participant felt that the term “disease” had negative connotations that may be offensive to some patients. The potential benefits of classifying obesity as a disease aligned with the TDF domain of “memory, attention, and decision processes.” Participants unanimously agreed that a clinical diagnosis could enhance their decision‐making abilities. By framing obesity as a disease, GPs expressed increased confidence in developing tailored care plans and accessing necessary resources. Aligned with the TDF domain of “social influence,” participants recognized their role in reframing obesity from a lifestyle consequence to a complex disease. This shift in narrative, they believed, could significantly improve patient outcomes. As one GP expressed,: *As healthcare professionals, if we start talking about it more as a disease and framing it that way, it would be much more helpful to patients*. *(GP,M*).

### 8.2 Inherent lifestyle biases

The domains “knowledge” and “beliefs about consequences” were closely aligned. Participants generally attributed obesity to lifestyle factors while acknowledging its complexity beyond simple dietary and exercise modifications. Although explicit biases were not expressed, underlying assumptions about causal factors were evident. As one GP described: *I think there's still a little bit of old world thinking […] and some of the negative connotations, attitudes out there, and so on. And people regarding it too simplistically, it's just a function of diet and exercise. (GP,M*).

## 9 BELIEFS ABOUT CAPABILITIES

### 9.1 Competency beliefs—not equipped to do it alone

This theme reflected findings in *Knowledge* and *Skills*. Participants expressed a lack of confidence in managing obesity independently within general practice, highlighting the need for enhanced knowledge, skills, and allied health support. Participants emphasized the complexity of obesity diagnosis, criticizing the limitations of body mass index (BMI) as a sole diagnostic tool. They argued that the multifaceted nature of obesity demands a more nuanced approach, akin to other chronic conditions with clearly defined diagnostic criteria and objective biomarkers: *This isn't a one size fits all, here's your checklist […] I'll check your BMI, blood pressure […] fasting lipids. […] Job done. It's just not going to work like that. (GP,F*).

## 10 REINFORCEMENT

### 10.1 Defaulting to ‘eat less, move more’

The absence of clear clinical protocols and limited access to resources compelled GPs to rely on oversimplified lifestyle advice as the primary approach to weight management. Despite growing recognition of obesity's complexity, the “eat less, move more” paradigm persisted as the default treatment strategy. As one GP stated: *We possibly are more accepting of the fact that there is now a science…but how we address the issue hasn't changed hugely…it's still the input, output balance. (GP,M*).

A lack of support further hindered the initiation of more comprehensive treatment plans. While GPs acknowledged the limitations of traditional approaches and the need for adjunctive interventions, they expressed reluctance to initiate alternative treatments due to knowledge gaps and insufficient access to allied health services. As one GP described: *The challenge comes when I have a patient sitting in front of me that Ive got to the end of my skill set, who has nothing more to, you know, Ive nothing more to offer, but there's nobody else available to me […] I'm hesitant to start that pathway if I don't know what the safety net is […] because it's not fair on the patient (GP,F*).

## 11 SOCIAL, PROFESSIONAL ROLE AND IDENTITY

### 11.1 Role expectation and responsibility

Participants expressed ambivalence about their role in prescribing novel pharmacological interventions for obesity. While recognizing potential benefits, they questioned their capacity to effectively manage these medications without adequate support, including nutrition, psychological, and physiotherapy services. The traditional “doctor as healer” model was deemed insufficient for addressing such a complex condition. Instead, GPs envisioned a collaborative approach involving patients and other healthcare professionals to develop personalized treatment plans. *[GPs] play a role in monitoring certain parameters, and possibly in prescribing but we certainly can't do it all, […] we will only ever seem to have parts of those jigsaws in place. We never seem to have all of the component pieces. (GP,F*).

### 11.2 Not consulted as integral stakeholders

Participants reported feeling excluded from the policy‐making process for obesity management. Despite occasional consultation, their insights were often overlooked, leading to policies perceived as unrealistic and impractical for general practice. This disconnect between policy and practice fostered a pessimistic outlook among the participants regarding potential system‐wide improvements in obesity care: *We don't generally feel very consulted, [policies] are generally told to us rather than that we are asked for input in a meaningful way, and very often where we are asked for input, there's a bit of cherry picking […] but, the problem is that the resourcing doesn't follow. (GP, F)*.

## 12 SOCIAL INFLUENCES

### 12.1 Peer education

The participants expressed a strong desire for ongoing education and training in obesity management While recognizing the availability of self‐directed learning opportunities, participants emphasized the need for structured, targeted training. They advocated for a more proactive role from the GP representative body in ensuring access to obesity‐specific training. Participants emphasized a preference for peer education, specifically from GPs with expertise in obesity management. This preference stemmed from the belief that colleagues with an understanding of the daily challenges faced by GPs would be more effective in delivering impactful training: *There's a combined responsibility here from our representative organization with the department of health to actually say, are there [obesity specialist GPs] in the country who get what is to be a GP and understands what we are and can help put something together that will actually work. (GP, F)*.

## 13 BEHAVIORAL REGULATION

### 13.1 Actionable insights

Time constraints were identified as a key barrier to professional development. Participants emphasized the need for practical and easily accessible support materials that could be integrated into existing workflows. Flexible online modules were suggested as a potential solution, allowing GPs to engage with content in short, manageable bursts: *I think webinars have been great, because you can do it in your own time. I think the online modules can be really helpful, you know. Just like short snippets so you can do 10, 20* *min here and there, work it around your schedule. (GP,F).* Participants also advocated for integrating obesity‐related services into existing electronic referral systems to streamline the referral process and improve continuity of care.

## 14 IDENTIFYING INTERVENTION TARGETS

The findings were mapped onto the COM‐B model to identify potential intervention functions and policy categories using the BCW to guide the process. Six out of possible nine intervention functions were deemed relevant based on the outcome of the COM‐B mapping, as shown in Table 1. The intervention functions included; *Education* (defined as increasing knowledge and understanding), *Persuasion* (defined as using communication to induce positive or negative feelings to stimulate action), *Environmental restructuring* (defined as changing the physical or social environment), *Enablement* (defined as increasing means/reducing barriers to increase capability), *Modeling* (defined as providing an example for people to aspire to or imitate) and, *Training* (defined as imparting skills). Four policy categories were identified that could support the delivery of the intervention functions.

**TABLE 1:** Combined link between COM‐B and TDF domains, intervention functions, policy categories, BCTs, and suggested intervention strategies.

TDF domains (COM‐B)	Indicated intervention functions	Policy categories	Behavior change techniques (BCTs)	Example of potential strategies
Social, professional role and identity (Ref M)	Education, persuasion	Guidelines, service provision	‐‐‐‐‐‐	‐‐‐‐‐‐
Environment, context and resources (Phys O)	Environmental restructuring, enablement	Guidelines, service provision	12.5.Adding objects to the environment	Provide clinical practice protocols
Beliefs about consequences (Ref M)	Education, persuasion	Communication/marketing, guidelines, service provision	5.1.Information about health consequences, 5.6.Information about emotional consequences, 9.2.Pros and cons, 13.1.Identification of self as role model	Provide information on the impact of weight bias on patient outcomes
Beliefs about capabilities (Ref M)	Education, training, enablement	Guidelines, service provision	4.1.Instruction on how to perform a behavior, 6.1.Demonstration of behavior, 8.1.Behavioral practice/rehearsal 15.1.Verbal persuasion about capability	Training on communication skills, provide education on the etiology of obesity and evidence‐based treatment strategies
Knowledge (Psy C)	Education	Communication/marketing, guidelines	4.1.Instructions on how to perform a behavior	Provide education on the etiology of obesity and evidence‐based treatment strategies
Social influences (Soc O)	Modeling	Communication/marketing, service provision	3.2.Social support (practical), 6.2.Social comparison, 9.1.Credible source	In‐group subject expert to promote evidence informed practice
Skills (Phys C)	Training	Guidelines, service provision	4.1.Instruction on how to perform a behavior, 6.1.Demonstration of behavior, 8.1.Behavioral practice/rehearsal, 8.3.Habit formation	Training on communication skills
Optimism (Ref M)	Persuasion	Service provision	‐‐‐‐‐	‐‐‐‐‐
Emotion (Auto M)	Enablement, persuasion	Guidelines, service provision	5.5.Anticipated regret, 11.2.Reduce negative emotions, 13.2. Framing/reframing	Teach emotional regulation strategies to manage negative emotions (cognitive reappraisal)
Reinforcement (Auto M)	Environmental restructuring	Guidelines, service provision	12.5.Adding objects to the environment	Provide clinical practice protocols
Memory, attention and decision making (Psy C)	Environmental restructuring	Guidelines	12.5.Adding objects to the environment	Provide clinical practice protocols
Behavioral regulation (Psy C)	Education, training, enablement	Guidelines, environmental/social planning	1.4.Action planning, 4.1.Instruction on how to perform a behavior, 6.1.Demonstration of behavior, 7.1.Prompts/cues, 8.1.Behavioral practice/rehearsal, 8.3.Habit formation, 12.5.Adding objects to the environment,	Provide education on the etiology of obesity and evidence‐based treatment strategies

*Note*: COM‐B components; (Psy C), psychological capability; (Phys C), psychical capability; (Phys O), physical opportunity; (Soc O), social opportunity; (Ref M), reflective motivation; (Auto M), automatic motivation. Behavioral change techniques; (BCTs).

The policy functions included *guidelines* (for instance, the creation and dissemination of documents that mandate practice and treatment protocols), *service provision* (establishing support services in the community), *communication/marketing* (examples of which include the delivery of on‐line webinars to provide obesity education and training to GPs), and *environmental/social planning* (examples include the introduction of formal education on obesity as a disease in medical school and GP training programmes). Behavior change techniques (BCTs) are the active ingredients empirically shown to promote behavior modification within health interventions. Eighteen BCTs from the BCT Taxonomy Version 1.^20^ were linked to the qualitative findings and subsequent behavioral diagnosis. These results are detailed in Table 1. The implications of these findings are discussed below.

## 15 DISCUSSION

### 15.1 Principal findings

Findings highlighted a lack of knowledge of obesity as a disease as a key factor in current obesity‐related clinical practices. This is in contrast to the dominant quantitative literature where it was reported that obesity is widely accepted as a disease by healthcare providers.^32^, ^33^ Similar to previous research, knowledge gaps were reflected in the participants belief that obesity was a consequence of lifestyle factors and considered refractory to treatment.^34^, ^35^ Additional insights revealed that knowledge gaps impacted participants' beliefs about their capability to manage the complexity of obesity in isolation in general practice, specifically when navigating the diagnosis, and the prescription of novel pharmacological interventions. This was magnified by the perceived absence of clear diagnostic criteria and clinical treatment protocols. These knowledge gaps resulted in practitioners resorting to oversimplified advice related to lifestyle factors as causal determinates and treatment approaches for the disease.^6^, ^36^

A key contextual factor contributing to a poor understanding of obesity‐related clinical practice was that obesity was not prioritized in medical education at undergraduate, postgraduate or in continuous professional development.^7^, ^37^ Obesity was presented as a risk factor for comorbid diseases with latent connotations of it being self‐inflicted due to its association with poor nutrition and physical inactivity. This oversimplified presentation of obesity has real‐life implications for PwO. Most notable is recent research reporting that patients in receipt of oversimplified approaches to obesity management felt weight‐stigmatized, a known contributing factor to comorbid health complications and reductions in healthcare utilization.^35^, ^38^, ^39^, ^40^ GPs reported being aware that obesity was more complex than “eat less, move more;” however, in the absence of understanding the complexity of the disease, and knowledge of adjunctive treatment approaches, they did not feel competent to treat obesity in isolation in general practice.

Similar to previous findings, the participants indicated a tendency to circumvent weight‐based conversations due to their limited knowledge of obesity management and fear of offending the patient.^4^, ^41^ This study provides more nuance with GPs, indicating that the primary barrier was a skills deficit in knowing *how* and *when* to initiate the conversation, thus suggesting that GPs may benefit from explicit weight‐based conversation training. In combination, these findings reflect the broader literature indicating a critical need for contemporary obesity education and skills training to be integrated into medical school curricula, postgraduate training programmes, and continuous professional development.^7^, ^36^, ^42^, ^43^, ^44^

Challenges were also perceived at the boarder service level, most notably a lack of timely access to allied health services to support and optimize obesity management. Resourcing is required to provide meaningful support for patients living with obesity.^44^ Consistent with previous research, there was a general pessimism due to extrinsic factors that influenced how GPs felt they were valued in the healthcare system.^45^ In particular, there was a perceived disconnect in communication between GPs and policy makers. GPs felt that generally they were not consulted in the drafting of health policies, which resulted in proposals being perceived as “*not fit for purpose*.” This perceived disconnect between government and general practice was reflected in the fact that most of the GPs reported having limited or no knowledge of the proposed MOC for Obesity, revealing an important barrier to its adoption and implementation.

### 15.2 Implications for future intervention development

The BCW indicated education and training as intervention functions to target knowledge gaps and perceived skills deficits (Table 1). These intervention functions were also identified for TDF domains: beliefs about consequences, beliefs about capabilities and behavioral regulation. Thus, these strategies will be crucial in addressing key mechanisms of change to improve obesity‐related clinical practices. This could involve the provision of empirical evidence to increase understanding of obesity as a disease and increase awareness of evidence‐based interventions to support the management of obesity in general practice. Evidence from previous research has demonstrated that continuing medical education has increased knowledge, optimized prescriptions and improved overall patient outcomes.^46^, ^47^, ^48^ Similarly, several reviews reported that communication skills training enhanced primary healthcare professionals knowledge, boosted competency beliefs, and ultimately contributed to improved patient outcomes.^49^, ^50^, ^51^

The BCW identified resourcing and structural factors as key targets for intervention, suggesting strategies such as environmental restructuring, enablement, and training. For example, providing GPs with advanced training through online educational webinars could be beneficial. The findings highlighted the need for structural changes to current clinical pathways, necessitating changes to the surrounding policy environment (Table 1) to facilitate clinical re‐structuring and optimize obesity‐related clinical practices.^44^

Future interventions will benefit from the evaluation of the indicated intervention functions, policy categories and suggested BCTs with integral stakeholders to ascertain the most practical BCTs and mode of delivery to apply.^19^ It is recommended that obesity‐related health policies directed at GPs be co‐developed with GPs and effectively communicated to promote adoption and implementation. Finally, given the polarity on the participants understanding of obesity as a disease compared to the quantitative literature,^32^, ^33^ more qualitative research is recommended to ensure that all barriers to the provision of quality care for obesity in general practice are identified.

### 15.3 Strengths & limitations

This study pioneered the use of the TDF and BCW to explore factors influencing GPs' obesity‐related clinical practices. By combining these frameworks, a comprehensive understanding of the complex interplay between knowledge, beliefs, and environmental factors shaping GP behavior was achieved. This research fills a critical gap in the literature by providing a theoretical foundation for future intervention development targeting obesity management in general practice. The study findings may not be fully generalizable beyond the Irish general practice context due to potential variations in healthcare systems and GP roles. While the small sample size is a limitation, it is comparable to other studies recruiting GPs and reflects the challenges of participant recruitment in this population.^52^ Nonetheless, the study's rigorous methodology, including the use of established theoretical frameworks and in‐depth analysis, strengthens the validity of the findings for the study.^30^

## 16 CONCLUSIONS

This study provided a comprehensive insight into the key behavioral and contextual influences on GPs obesity‐related clinical practices. The application of the TDF and the BCW identified a complex interplay of knowledge gaps, beliefs and environmental factors shaping GP behavior. To address these issues, future interventions should prioritize education, training, and practice environment enhancements. Developing and disseminating clinical guidelines and treatment protocols alongside accessible training programmes is essential for improving obesity care. Additionally, co‐developing interventions with GPs will facilitate their adoption and implementation.

## AUTHOR CONTRIBUTIONS

LR conceived the study, collected the data, analysed the data and led the writing of the manuscript. MH co‐analysed the data. G’OD, CH, JW, OC, SB and MC were involved in the interpretation of the data, writing the paper, and final approval of the submitted and published versions.

## CONFLICT OF INTEREST STATEMENT

Michael Crotty reports honoraria for educational events or conference attendance from Novo Nordisk and Consilient Health and was a member of the Novo Nordisk advisory board. He is a member of the Irish ONCP Clinical Advisory Group and ASOI. Michael is the co‐founder and clinical lead of “My Best Weight Clinic.” Susie Birney reports funding to ICPO from the HSE, Novo Nordisk, and the European Coalition for People Living with Obesity (ECPO) and consulting fees or honoraria from Diabetes Ireland, ECPO, and Novo Nordisk. Susie is the Executive Director of ICPO and the Secretary of ECPO.

## Supporting information

Supporting Information S1

## References

[1] Lobstein T , Brinsden H , Neveux M . World obesity atlas 2022. World Obesity Federation; 2022. https://policycommons.net/artifacts/2266990/world_obesity_atlas_2022_web/3026660/?utm_medium=email&utm_source=transaction

[2] Keaver L , Webber L , Dee A , et al. Application of the UK foresight obesity model in Ireland: the health and economic consequences of projected obesity trends in Ireland. PLoS One. 2013;8(12):e79827. 10.1371/journal.pone.0079827 24236162 PMC3827424

[3] Health Service Executive (HSE) . Model of Care for the Management of Overweight and Obesity. Royal College of Physicians in Ireland; 2021.

[4] Tremblett M , Poon AYX , Aveyard P , Albury C . What advice do general practitioners give to people living with obesity to lose weight? A qualitative content analysis of recorded interactions. Fam Pract. 2022;XX(5‐6):1‐7. 10.1093/fampra/cmac137 PMC10745272 36510443

[5] Yunus NA , Russell G , Muhamad R , Soh S.‐E , Sturgiss E . The perceptions of healthcare practitioners on obesity management in Peninsular Malaysia: a cross‐sectional survey. BMC Health Serv Res. 2023;23(1):744. 10.1186/s12913-023-09759-z 37430243 PMC10334633

[6] Booth HP , Prevost AT , Gulliford MC . Access to weight reduction interventions for overweight and obese patients in UK primary care: population‐based cohort. BMJ Open. 2015;5(1):e006642. 10.1136/bmjopen-2014-006642 PMC4316417 25586371

[7] Mastrocola MR , Roque SS , Benning LV , Stanford FC . Obesity education in medical schools, residencies, and fellowships throughout the world: a systematic review. Int J Obes. 2019;44(2):269‐279. 10.1038/s41366-019-0453-6 PMC7002222 31551484

[8] Wangler J , Jansky M . Attitudes, behaviours and strategies towards obesity patients in primary care: a qualitative interview study with general practitioners in Germany. Eur J Gen Pract. 2021;27(1):27‐34. 10.1080/13814788.2021.1898582 33749477 PMC7993392

[9] Epstein L , Ogden J . A qualitative study of GPs' views of treating obesity. Br J Gen Pract. 2005;55(519):750‐754. 16212849 PMC1562352

[10] Malterud K , Ulriksen K . Obesity, stigma, and responsibility in health care: a synthesis of qualitative studies. Int J Qual Stud Health Well‐Being. 2011;6(4):8404. 10.3402/qhw.v6i4.8404 PMC3223414 22121389

[11] Puhl RM , Lessard LM , Himmelstein MS , Foster GD . The roles of experienced and internalized weight stigma in healthcare experiences: perspectives of adults engaged in weight management across six countries. PLoS One. 2021;16(6):e0251566. 10.1371/journal.pone.0251566 34061867 PMC8168902

[12] Ryan L , Quigley F , Birney S , Crotty M , Conlan O , Walsh J . Beyond the scale’: a qualitative exploration of the impact of weight stigma experienced by patients with obesity in general practice. Health Expect. 2024;27(3):e14098. 10.1111/hex.14098 38859797 PMC11165259

[13] Breen C , O’Connell J , Geoghegan J , et al. Obesity in adults: a 2022 adapted clinical practice guideline for Ireland. Obes Facts. 2022;15(6):736‐752. 10.1159/000527131 36279848 PMC9801383

[14] Schwenke M , Luppa M , Pabst A , et al. Attitudes and treatment practice of general practitioners towards patients with obesity in primary care. BMC Fam Pract. 2020;21(169):169. 10.1186/s12875-020-01239-1 32807094 PMC7433134

[15] Gardner B , Richards R , Lally P , Rebar A , Thwaite T , Beeken RJ . Breaking habits or breaking habitual behaviours? Old habits as a neglected factor in weight loss maintenance. Appetite. 2021;162:e105183. 10.1016/j.appet.2021.105183 33651994

[16] Atkins L , Francis J , Islam R , et al. A guide to using the Theoretical Domains Framework of behaviour change to investigate implementation problems. Implement Sci. 2017;12(1):77. 10.1186/s13012-017-0605-9 28637486 PMC5480145

[17] Colquhoun HL , Brehaut JC , Sales A , et al. A systematic review of the use of theory in randomized controlled trials of audit and feedback. Implement Sci. 2013;8(1):66. 10.1186/1748-5908-8-66 23759034 PMC3702512

[18] Cane J , O’Connor D , Michie S . Validation of the theoretical domains framework for use in behaviour change and implementation research. Implement Sci. 2012;7(1):1‐17. 10.1186/1748-5908-7-37 PMC3483008 22530986

[19] Michie S , van Stralen MM , West R . The behaviour change wheel: a new method for characterising and designing behaviour change interventions. Implement Sci. 2011;6(1):42. 10.1186/1748-5908-6-42 21513547 PMC3096582

[20] Michie S , Richardson M , Johnston M , et al. The behaviour change technique taxonomy (v1) of 93 hierarchically clustered techniques: building an international consensus for the reporting of behaviour change interventions. Ann Behav Med. 2013;46(1):81‐95. 10.1007/s12160-013-9486-6 23512568

[21] Wong E , Mavondo F , Horvat L , McKinlay L , Fisher J . Healthcare professionals' perspective on delivering personalised and holistic care: using the Theoretical Domains Framework. BMC Health Serv Res. 2022;22(1):281. 10.1186/s12913-022-07630-1 35232432 PMC8887936

[22] Bradley CP , Byrne M , Payne RA , Duerden M , Byrne M . Improving medication management in multimorbidity: development of the MultimorbiditY COllaborative medication review and DEcision making (MY COMRADE) intervention using the behaviour change wheel. Implement Sci. 2015;10(1):132. 10.1186/s13012-015-0322-1 26404642 PMC4582886

[23] Cadogan SL , McHugh SM , Bradley CP , Browne JP , Cahill MR . General practitioner views on the determinants of test ordering: a theory‐based qualitative approach to the development of an intervention to improve immunoglobulin requests in primary care. Implement Sci. 2016;11(1):102. 10.1186/s13012-016-0465-8 27435839 PMC4952272

[24] Tong A , Sainsbury P , Craig J . Consolidated criteria for reporting qualitative research. Int J Qual Health Care. 2007;19(6):349‐357. 10.1093/intqhc/mzm042 17872937

[25] Thompson J . A guide to abductive thematic analysis. Qual Rep. 2022;27(5):1410‐1421. 10.46743/2160-3715/2022.5340

[26] Kaushik V , Walsh CA . Pragmatism as a research paradigm and its implications for social work research. Soc Sci. 2019;8(9):255. 10.3390/socsci8090255

[27] Hurley E , Dietrich T , Rundle‐Thiele S . Integrating theory in Co‐design: an abductive approach. Australas Market J. 2021;1:66‐77. 10.1177/1839334921998541

[28] Mantell R , Withall A , Radford K , Kasumovic M , Monds L , Hwang YIJ . Design preferences for a serious game–based cognitive assessment of older adults in prison: thematic analysis. JMIR Serious Games. 2023;11:e45467. 10.2196/45467 37067850 PMC10152383

[29] Malterud K , Siersma VD , Guassora AD . Sample size in qualitative interview studies: guided by information power. Qual Health Res. 2016;26(13):1753‐1760. 10.1177/1049732315617444 26613970

[30] McGowan LJ , Powell R , French DP . How can use of the Theoretical Domains Framework be optimized in qualitative research? A rapid systematic review. Br J Health Psychol. 2020;25(3):677‐694. 10.1111/bjhp.12437 32558289

[31] Nvivo Qualitative Data Analysis Version 1.0 (software). QSR International Pty Ltd; 2020.

[32] Sharma AM , Bélanger A , Carson V , et al. Perceptions of barriers to effective obesity management in Canada: results from the ACTION study. Clinical Obesity. 2019;9(5):e12329. 10.1111/cob.12329 31294535 PMC6771494

[33] Kaplan LM , Golden A , Jinnett K , et al. Perceptions of barriers to effective obesity care: results from the national ACTION study. Obesity. 2018;26(1):61‐69. 10.1002/oby.22054 29086529

[34] O’Keeffe M , Flint SW , Watts K , Rubino F . Knowledge gaps and weight stigma shape attitudes toward obesity. Lancet Diabetes Endocrinol. 2020;8(5):363‐365. 10.1016/s2213-8587(20)30073-5 32142624

[35] O’Donoghue G , Cunningham C , King M , O’Keefe C , Rofaeil A , McMahon S . A qualitative exploration of obesity bias and stigma in Irish healthcare; the patients' voice. PLoS One. 2021;16(11):e0260075. 10.1371/journal.pone.0260075 34843517 PMC8629268

[36] Nagpal TS , Pearce N , Dhaliwal K , Abraham JR . Implementing evidence‐based obesity management guidelines requires development of medical competencies: a commentary outlining future directions in obesity education in Canada. Obesity Pillars. 2023;8:e100086. 10.1016/j.obpill.2023.100086 PMC10728703 38125664

[37] Butsch WS , Kushner RF , Alford S , Smolarz BG . Low priority of obesity education leads to lack of medical students’ preparedness to effectively treat patients with obesity: results from the U.S. medical school obesity education curriculum benchmark study. BMC Med Educ. 2020;20(1):1‐6. 10.1186/s12909-020-1925-z PMC6988262 31992274

[38] Ryan L , Coyne R , Heary C , et al. Weight stigma experienced by patients with obesity in healthcare settings: a qualitative evidence synthesis. Obes Rev. 2023;1(10):e13606. 10.1111/obr.13606 37533183

[39] Alberga AS , Edache IY , Forhan M , Russell‐Mayhew S . Weight bias and health care utilization: a scoping review. Prim Health Care Res Dev. 2019;1:e20. 10.1017/S1463423619000227 PMC6650789 32800008

[40] Daly M , Sutin AR , Robinson E . Perceived weight discrimination mediates the prospective association between obesity and physiological dysregulation: evidence from a population‐based cohort. Psychol Sci. 2019;30(7):1030‐1039. 10.1177/0956797619849440 31158067 PMC6657150

[41] Blackburn M , Stathi A , Keogh E , Eccleston C . Raising the topic of weight in general practice: perspectives of GPs and primary care nurses. BMJ Open. 2015;8:e008546. 10.1136/bmjopen-2015-008546 PMC4538258 26254471

[42] Stanford FC , Johnson ED , Claridy MD , Earle RL , Kaplan LM . The role of obesity training in medical school and residency on bariatric surgery knowledge in primary care physicians. Int J Family Med. 2015;2015:841249. 10.1155/2015/841249 26339506 PMC4539067

[43] Vitolins MZ , Crandall S , Miller D , Ip E , Marion G , Spangler JG . Obesity educational interventions in U.S. Medical schools: a systematic review and identified gaps. Teach Learn Med. 2012;24(3):267‐272. 10.1080/10401334.2012.692286 22775792 PMC3811015

[44] Breen C , O’Connell J , Geoghegan J , et al. Obesity in adults: a 2022 adapted clinical practice guideline for Ireland. Obes Facts. 2022;15(6):736‐752. 10.1159/000527131 36279848 PMC9801383

[45] Tanne JH . Gp’s in 10 prosperous countries feel undervalued and overworked, survey finds. BMJ. 2023;382:1925. 10.1136/bmj.p1925 37604579

[46] Thepwongsa I , Kirby CN , Schattner P , Piterman L . Online continuing medical education (CME) for GPs: does it work? A systematic review. Aust Fam Physician. 2014;43(10):717‐721. 25286431

[47] Mostofian F , Ruban C , Simunovic N , Bhandari M . Changing physician behaviour: what works. Am J Manag Care. 2015;21(1):75‐84. 25880152

[48] Thomas DC , Johnston B , Dunn K , et al. Continuing medical education, continuing professional development, and knowledge translation: improving care of older patients by practicing physicians. J Am Geriatr Soc. 2006;54/10(10):1610‐1618. 10.1111/j.1532-5415.2006.00879.x 17038082

[49] Chauhan BF , Jeyaraman M , Mann AS , et al. Behaviour change interventions and policies influencing primary healthcare professionals’ practice—an overview of reviews. Implement Sci. 2017;12(1):3. 10.1186/s13012-016-0538-8 28057024 PMC5216570

[50] Moore PM , Rivera Mercado S , Grez Artigues M , Lawrie TA . Communication skills training for healthcare professionals working with people who have cancer. Cochrane Database Syst Rev. 2013;3:CD003751. 10.1002/14651858.cd003751.pub3 PMC6457800 23543521

[51] Sikorski C , Luppa M , König H.‐H , van den Bussche H , Riedel‐Heller SG . Does GP training in depression care affect patient outcome? ‐ a systematic review and meta‐analysis. BMC Health Serv Res. 2012;12(1):10. 10.1186/1472-6963-12-10 22233833 PMC3266633

[52] McKinn S , Bonner C , Jansen J , McCaffery K . Recruiting general practitioners as participants for qualitative and experimental primary care studies in Australia. Aust J Prim Health. 2015;21(3):354‐359. 10.1071/py14068 25074256

