# Supplementary material for: Factors that influence general practitioners' obesity‐related clinical practices and determinants of behavior to target to promote best practice in obesity care: A qualitative exploration
Source: Obes Sci Pract. 2024 Sep 28;10(5):e70012. doi: 10.1002/osp4.70012 (PMC11438194; doi:10.1002/osp4.70012)
Supplement: Supplementary file 1 — Supporting Information S1 [file OSP4-10-e70012-s001.pdf]

Supporting Information

Title: Factors that influence general practitioners' obesity-related clinical practices and determinants of behaviour to target to promote best practice in obesity care: a qualitative exploration

Author(s): Leona Ryan[1], Grainne O'Donoghue[2], Caroline Heary[1], Susie Birney[4], Michael Crotty[5], Michelle Hanlon[1], Owen Conlan[3], Jane C. Walsh[1]

Affiliation(s) [1]School of Psychology, University of Galway, Ireland

[2]School of Public Health, Physiotherapy and Sports Science, University College Dublin, Ireland

[3]School of Computer Science and Statistics, Trinity College Dublin, Ireland

[4]Irish Coalition for People Living with Obesity (ICPO), Ireland

[5] Irish College of General Practitioners (ICGP), Clinical Lead Obesity, Ireland

Key words: obesity, general practice, behaviour change, qualitative

Running title: GPs obesity-related clinical practices

Acknowledgements: LR is in receipt of the following financial support for the research, authorship, and/or publication of this article: This work was supported by the Science Foundation Ireland Centre for Research Training in Digitally-Enhanced Reality (D-REAL) under Grant No.18/CRT/6224

Corresponding author: Leona Ryan, School of Psychology, University of Galway, Ireland, H91 TK33. Email: L.ryan33@universityofgalway.ie Tel: +00353879330541

Supplementary Information 1, Table 1

Table 1: Consolidated criteria for reporting qualitative studies (COREQ): 32-item checklist.

No. Item	Guide questions/description	Reported on Page #
**Domain 1: Research team and reflexivity**		
Personal Characteristics		
1. Interviewer/facilitator	Which author/s conducted the interview or focus group?	Pg 6, L5
2. Credentials	What were the researcher's credentials? E.g. PhD, MD	Pg 4, L6-9
3. Occupation	What was their occupation at the time of the study?	Page 4, L6-9
4. Gender	Was the researcher male or female?	Pg 6, L12
5. Experience and training	What experience or training did the researcher have?	Pg 5, L6-9
Relationship with participants		
6. Relationship established	Was a relationship established prior to study commencement?	No
7. Participant knowledge of the interviewer	What did the participants know about the researcher? e.g. personal goals, reasons for doing the research	Participants were briefed on the purpose of the study via the participant information sheet administered prior to them giving informed consent to participate.
8. Interviewer characteristics	What characteristics were reported about the inter viewer/facilitator? e.g., Bias, assumptions, reasons, and interests in the research topic	Pg 4, L6-9

Supplementary Information 1, Table 1 cntd.

**Domain 2: study design**		
Theoretical framework		
9. Methodological orientation and Theory	What methodological orientation was stated to underpin the study? e.g. grounded theory, discourse analysis, ethnography, phenomenology, content analysis	Pg 4, L20-29
Participant selection		
10. Sampling	How were participants selected? e.g. purposive, convenience, consecutive, snowball	Pg 5, L2-7
11. Method of approach	How were participants approached? e.g. face-to-face, telephone, mail, email	Pg 5, L2-7
12. Sample size	How many participants were in the study?	Pg 5, L6-7
13. Non-participation	How many people refused to participate or dropped out? Reasons?	None
Setting		
14. Setting of data collection	Where was the data collected? e.g. home, clinic, workplace	Online, via Zoom. Pg 5, L21
15. Presence of non-participants	Was anyone else present besides the participants and researchers?	No
16. Description of sample	What are the important characteristics of the sample? e.g. demographic data, date	Pg 5, Table 1
Data collection		
17. Interview guide	Were questions, prompts, guides provided by the authors? Was it pilot tested?	Pg 5, L16-20
18. Repeat interviews	Were repeat inter views carried out? If yes, how many?	No
19. Audio/visual recording	Did the research use audio or visual recording to collect the data?	Pg 5, L21 – Pg6, L2
20. Field notes	Were field notes made during and/or after the interview or focus group?	Pg 6, L2
21. Duration	What was the duration of the inter views or focus group?	Pg 6, L1-2
22. Data saturation	Was data saturation discussed?	Pg 5, L7-10
23. Transcripts returned	Were transcripts returned to participants for comment and/or correction?	No

Supplementary Information 1, Table 1 cntd.

**Domain 3: analysis and findings**		
Data analysis		
24. Number of data coders	How many data coders coded the data?	LR coded the initial data, the second round of coding was in collaboration with MH.
	Did authors provide a description of the coding tree?	Pg 6, L10-16
26. Derivation of themes	Were themes identified in advance or derived from the data?	Pg 6, L9-20
27. Software	What software, if applicable, was used to manage the data?	Nvivo 20
28. Participant checking	Did participants provide feedback on the findings?	No
Reporting		
29. Quotations presented	Were participant quotations presented to illustrate the themes/findings? Was each quotation identified? e.g. participant number	Yes, all findings are supported with primary data.
30. Data and findings consistent	Was there consistency between the data presented and the findings?	Yes
31. Clarity of major themes	Were major themes clearly presented in the findings?	Yes
32. Clarity of minor themes	Is there a description of diverse cases or discussion of minor themes?	Yes

Supplementary information 2, Table 2

Table 2: TDF domains; definitions and constructs in each domain (Atkins et al., 2017)

TDF Domain(s)	Definition	Constructs
1. Knowledge	An awareness of the existence of something	Knowledge (including knowledge of condition/scientific rationale)<br>Procedural knowledge<br>Knowledge of task environment
2. Skills	An ability or proficiency acquired through practice	Skills<br>Skills development<br>Competence<br>Ability<br>Interpersonal skills<br>Practice Skill assessment<br>Professional
3. Social/professional role and identity	A coherent set of behaviours and displayed personal qualities of an individual in a social or work setting	Professional identity<br>Professional role<br>Social identity<br>Identity<br>Professional boundaries<br>Professional confidence<br>Group identity<br>Leadership<br>Organisational commitment<br>Self-confidence
4. Beliefs about capabilities	Acceptance of the truth, reality or validity about an ability, talent, or facility that a person can put to constructive use	Self-confidence<br>Perceived competence<br>Self-efficacy<br>Perceived behavioural control.<br>Beliefs<br>Self-esteem<br>Empowerment<br>Professional confidence
5. Optimism	The confidence that things will happen for the best or that desired goals will be attained	Optimism<br>Pessimism<br>Unrealistic optimism
6. Beliefs about consequences	Acceptance of the truth, reality, or validity about outcomes of a behaviour in each situation	Beliefs<br>Outcome expectancies<br>Characteristics of outcome expectancies<br>Anticipated regret<br>Consequents
7. Reinforcement	Increasing the probability of a response by arranging a dependent relationship, or contingency, between the response and a given stimulus	Rewards (proximal/distal, valued/not valued, probable/improbable)<br>Incentives<br>Punishment<br>Consequences<br>Reinforcement<br>Contingencies<br>Sanctions<br>Stability
8. Intentions	A conscious decision to perform a behaviour or a resolve to act in a certain way	Stability of intentions<br>Stages of change model<br>Transtheoretical model and stages of change
9. Goals	Mental representations of outcomes or end states that an individual wants to achieve	Goals (distal/proximal)<br>Goal priority<br>Goal/target setting<br>Goals (autonomous/controlled)<br>Action planning<br>Implementation intention

10. Memory, attention, and decision processes	The ability to retain information, focus selectively on aspects of the environment and choose between two or more alternatives	Memory Attention<br>Attention control<br>Decision making<br>Cognitive overload/tiredness
11. Environmental context and resources	Any circumstance of a person's situation or environment that discourages or encourages the development of skills and abilities, independence, social competence, and adaptive behaviour	Environmental stressors<br>Resources/material resources<br>Organisational culture/climate<br>Salient events/critical incidents<br>Person by environment interaction<br>Barriers and facilitators
12. Social influences	Those interpersonal processes that can cause individuals to change their thoughts, feelings, or behaviours)	Social pressure<br>Social norms<br>Group conformity<br>Social comparisons<br>Group norms<br>Social support<br>Power<br>Intergroup conflict<br>Alienation<br>Group identity<br>Modelling
13. Emotion	A complex reaction pattern, involving experiential, behavioural, and physiological elements, by which the individual attempts to deal with a personally significant matter or event	Fear<br>Anxiety<br>Affect<br>Stress<br>Depression<br>Positive/negative affect<br>Burnout
14. Behavioural regulation	Anything aimed at managing or changing objectively observed or measured actions	Self-monitoring<br>Breaking habit<br>Action planning

Source: 13. Atkins L, Francis J, Islam R, et al. A guide to using the Theoretical Domains Framework of behaviour change to investigate implementation problems. *Implementation Science.* 2017;12(1):77.

Supplementary Information 3: Figure 1

Figure 1. Overview of the abductive thematic analysis

Step 1: Transcription and familiarisation.
The lead author (LR) transcribed the collected data verbatim, actively read the transcriptions and took notes that identified potential patterns across the data.

Step 2: Coding
Inductive line-by-line coding at the semantic level was conducted by LR. Codes were consolidated, iteratively refined and organised into initial conceptual clusters.

Step 3: Codebook
A codebook was generated to provide structure to the coding process.

Step 4 & 5: Theme Development and theorising
The initial conceptual clusters were re-examined through the lens of the Theoretical Domains Framework, deductive changes were made to the coding structure by the lead (LR) author either though additions or updates to the codebook. The updated codebook was reviewed by a second author (MH), any discrepancies were resolved through discussion with the broader research team. This was an iterative process that resulted in the final presentation of themes.

Step 6: Data Display
A thematic map displaying the data codes that informed each theme was generated and provided to the research team for discussion. Any discrepancies that arose were resolved through discussion with the research team and updated by LR.

Step 7: Writing up
The findings were written up by the lead author (LR) using the domains of the TDF as headings for each theme. Illustrative quotations were included to provide empirical evidence of the theorisation. The research team provided feedback on the write up, suggested changed were made by the lead author (LR).
